# Fevers

**Published:** 1904-09-10

**Authors:** 


### FEVERS.

(*Concluded from page* 393.)

Small-pox.—It has been a frequent contention of anti-vaccinationists that the immunity from small-pox enjoyed by Germany was due, not to efficient protection by vaccination and revaccination, but to a more perfect system of isolation than we enjoy in England. Mr. Walter Long, therefore, sent Dr. Bruce Low recently to Germany to investigate this point. Dr. Low[31] visited ten of the principal German cities, including Berlin, and has issued his report to the Local Government Board. He finds that very little provision for small-pox cases is made in these cities, and in nearly every instance the isolation pavilion is part of the general hospital, and is usually supplied with food from the general kitchen. Soiled linen, after disinfection, is sent to the common laundry. Berlin, with a population just under 2,000,000, provides 12 beds. Nuremberg does the same, but has not had one case for the last eleven years. Munich has isolated seven cases in the last eight years. If a case does occur no panic arises. The immediate attendants are revaccinated again. The routine vaccination and revaccination to which other possible contacts have already been subjected is deemed sufficient for them. At every place careful inquiries were instituted, and it was found that the immunity of the population surrounding the hospital lay in the protected character of the German people. For us, because of our comparatively unprotected state, such a method of isolation would be fatal. In Liverpool[32] an epidemic has recently occurred. During the 16 months following September 1902 over 2,000 cases were recognised, of whom all but two were treated in isolation hospitals. To the prompt and energetic action of Dr. Hope, the Medical Officer of Health, and his assistants is ascribed the rapid extermination of the outbreak—rapid considering the large number of unvaccinated persons in Liverpool. Dr. Hope has prepared tables showing the favourable influence of vaccination in modifying the severity of cases. Mild cases occurring in vaccinated people were regarded as chicken-pox in many instances, while other sufferers were so little ill that they escaped medical observation altogether. These mild cases contributed to the spread of the disease. In France[33] every person living in the country, foreigner or Frenchman, must by law undergo triple vaccination, in the first, eleventh, and twenty-first years, and the production and distribution of vaccine lymph is strictly under public control. Vagrancy appears to play an important part in the introduction of small-pox. Dr. Armstrong,[34] the Medical Officer of Health for Newcastle-on-Tyne, has ascertained that in 57 districts out of 111 attacked the disease was introduced by vagrants. W. C. Paterson,[35] writing from Argentina, says that the Gran Chaco Indians from experience believe so thoroughly in vaccination that they fight furiously for precedence at the vaccinating table, vaccine lymph being scarce. He believes that vaccination at any time during small-pox incubation before the rash appears will modify it. A. C. Smith[36] has used antistreptococcic serum in six cases of small-pox, one being of a severe confluent type. Full vesication but no pustulation occurred in all. J. T. C. Nash[37] is of opinion that, judging cautiously from a study of a small number of cases only, red light has some influence in modifying the pustular stage. The nervous complications of small-pox are discussed by Aldrich,[38] who has collected an extensive bibliography on the subject. W. de Korte[39] describes some cases of Kaffir milk-pox or amaas—a disease which appears to closely resemble, but not to be identical with, small-pox.

[31] Lancet, April 23, p. 1135. [32] Ibid., March 26, p. 881. [33] Ibid, Feb. 6. [34] Brit. Med. Jour., April 23, p. 967. [35] Ibid., Feb. 27, p. 488. [36] Med. Rec., April 2. [37] Lancet, March 5. [38] Amer. Jour. Med. Sc, Feb. [39] Lancet, May 7.

